# Interactions Between Motor Thalamic Field Potentials and Single-Unit Spiking Are Correlated With Behavior in Rats

**DOI:** 10.3389/fncir.2020.00052

**Published:** 2020-08-13

**Authors:** Matt Gaidica, Amy Hurst, Christopher Cyr, Daniel K. Leventhal

**Affiliations:** ^1^Neuroscience Graduate Program, University of Michigan, Ann Arbor, MI, United States; ^2^Department of Neurology, University of Michigan, Ann Arbor, MI, United States; ^3^Department of Biomedical Engineering, University of Michigan, Ann Arbor, MI, United States; ^4^Parkinson Disease Foundation Research Center of Excellence, University of Michigan, Ann Arbor, MI, United States; ^5^Department of Neurology, VA Ann Arbor Health System, Ann Arbor, MI, United States

**Keywords:** thalamus, electrophysiology, field potentials, rats, motor control

## Abstract

Field potential (FP) oscillations are believed to coordinate brain activity over large spatiotemporal scales, with specific features (e.g., phase and power) in discrete frequency bands correlated with motor output. Furthermore, complex correlations between oscillations in distinct frequency bands (phase-amplitude, amplitude-amplitude, and phase-phase coupling) are commonly observed. However, the mechanisms underlying FP-behavior correlations and cross-frequency coupling remain unknown. The thalamus plays a central role in generating many circuit-level neural oscillations, and single-unit activity in motor thalamus (Mthal) is correlated with behavioral output. We, therefore, hypothesized that motor thalamic spiking coordinates motor system FPs and underlies FP-behavior correlations. To investigate this possibility, we recorded wideband motor thalamic (Mthal) electrophysiology as healthy rats performed a two-alternative forced-choice task. Delta (1–4 Hz), beta (13–30 Hz), low gamma (30–70 Hz), and high gamma (70–200 Hz) power were strongly modulated by task performance. As in the cortex, the delta phase was correlated with beta/low gamma power and reaction time. Most interestingly, subpopulations of Mthal neurons defined by their relationship to the behavior exhibited distinct relationships with FP features. Specifically, neurons whose activity was correlated with action selection and movement speed were entrained to delta oscillations. Furthermore, changes in their activity anticipated power fluctuations in beta/low gamma bands. These complex relationships suggest mechanisms for commonly observed FP-FP and spike-FP correlations, as well as subcortical influences on motor output.

## Introduction

Field potential (FP) oscillations are rhythmic fluctuations in extracellular potentials that emerge from, and may regulate (Anastassiou et al., 2010), neuronal dynamics over a large spatiotemporal scale (Fries, 2015). Various FP features including phase, amplitude, and frequency are correlated with sensorimotor phenomena (Friston et al., 2015; Armstrong et al., 2018; Pesaran et al., 2018). Delta (~1–4 Hz) oscillations are correlated with movement kinematics (Bansal et al., 2011), reaction time (RT, Lakatos et al., 2008; Stefanics et al., 2010; Hamel-Thibault et al., 2018), and sensory thresholds (Schroeder and Lakatos, 2009; Fiebelkorn et al., 2013). Beta oscillations (~13–30 Hz) in the cortex and basal ganglia are enhanced under several conditions including pre-movement hold periods (Donoghue et al., 1998; Saleh et al., 2010), isometric contractions (Baker et al., 1997), post-movement “rebound” (Pfurtscheller et al., 1996; Feingold et al., 2015), and parkinsonism (Brown, 2006; Mallet et al., 2008; Ellens and Leventhal, 2013). Beta power is also correlated with prolonged RTs (Leventhal et al., 2012; Khanna and Carmena, 2017; Shin et al., 2017; van Wijk, 2017; Torrecillos et al., 2018) and slowed movement (Pogosyan et al., 2009; Lofredi et al., 2019). Conversely, movement onset is associated with decreased beta and increased gamma (~60–100 Hz) power (Feingold et al., 2015; Tan et al., 2019; but see Leventhal et al., 2012). Nonetheless, the mechanisms by which FP features and behavior are correlated remain unclear.

In addition to correlations with behavior, FP oscillations exhibit complex spatiotemporal relationships with each other and single-unit activity. Oscillations of different frequencies are commonly coupled to each other, both within and between brain regions (Lakatos et al., 2005; Canolty et al., 2007). For example, delta phase is correlated with beta oscillation amplitude (Saleh et al., 2010; López-Azcárate et al., 2013; Arnal et al., 2015; Hamel-Thibault et al., 2018; Grabot et al., 2019), and beta phase is correlated with the amplitude of higher frequency oscillations (de Hemptinne et al., 2013; Meidahl et al., 2019). These complex correlations provide rich information regarding neural dynamics but make it difficult to distinguish cause from effect.

The thalamus is a central hub in nearly all motor, sensory, and associative circuits, and therefore well-positioned to regulate circuit-wide neuronal oscillations. Indeed, thalamocortical circuits generate or modulate many well-described FP oscillations including sleep spindles (Halassa et al., 2011; Mak-McCully et al., 2017), cortical slow (<1 Hz) oscillations (Neske, 2015), delta rhythms (Fogerson and Huguenard, 2016), alpha/mu (~8–15 Hz) rhythms (Saalmann et al., 2012; Crunelli et al., 2018), beta rhythms (Bastos et al., 2014), and gamma rhythms (McAfee et al., 2018). Though many of these studies focused on sensory regions, motor thalamic (Mthal) spiking is also phase-locked to delta oscillations under anesthesia (Nakamura et al., 2014). Modeling studies suggest that motor system beta oscillations could result from layer-specific thalamocortical inputs (Sherman et al., 2016; Reis et al., 2019), though mechanisms intrinsic to the basal ganglia are also proposed as “beta generators” (McCarthy et al., 2011; Tachibana et al., 2011; Mirzaei et al., 2017). The strong associations between thalamic activity and brain rhythms suggest that Mthal, which is reciprocally connected with motor and premotor cortices, mediates many FP-FP and FP-behavior correlations. The goal of this work is to determine correlations between single unit Mthal activity, FP oscillations, and behavior.

We previously identified two populations of Mthal units whose activity is correlated with distinct aspects of performance in a two-alternative forced-choice task (Gaidica et al., 2018). Because different FP features are also correlated with specific behavioral metrics, we hypothesized that these functionally defined single unit populations exhibit distinct spike-FP relationships. We found Mthal FP-behavior correlations largely concordant with observations in the motor cortex and the basal ganglia (Leventhal et al., 2012). Furthermore, functionally defined Mthal single unit populations were differentially entrained to the phase of delta oscillations and the amplitude envelopes of beta/low gamma (~50 Hz) oscillations. These results suggest mechanisms for FP-FP and FP-single unit interactions, with important implications for their functional interpretation.

## Materials and Methods

### Experimental Design

**Figure 1:**
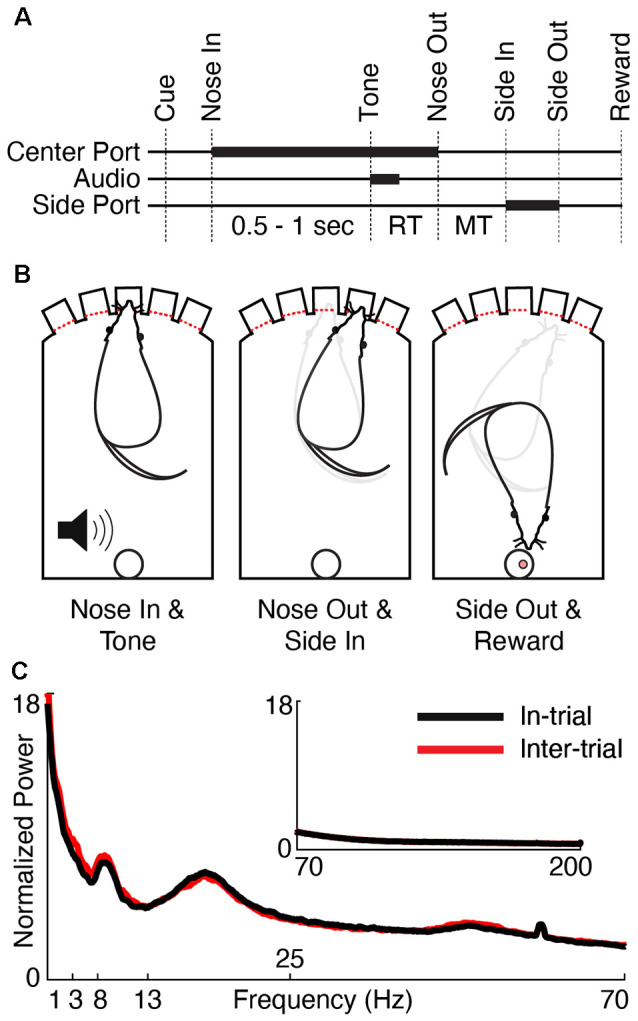
Behavioral task and physiology. **(A)** Trials began by illuminating one of the three center ports in a five-port behavior chamber (“Cue”). The rat poked and held its nose in the lit port (“Nose In”) for a variable interval (0.5–1 s, pulled from a uniform distribution) until a 1 or 4 kHz auditory cue (“Tone”) instructed the rat to move one port to the left or right, respectively. Nose Out, Side In, and Side-Out indicate when the rat withdrew from the central port, poked the adjacent port, and withdrew from the adjacent port, respectively. “Reward” indicates the time of reward pellet retrieval. Reaction time (RT) and movement time (MT) intervals are labeled. Thick lines indicate either nose port occupancy (Center and Side Port) or playing the auditory cue (Audio). **(B)** Schematic of the rat operant chamber during key behavioral epochs. **(C)** Session-averaged normalized power spectra (see “Materials and Methods” section, *Power Spectrum*) of low (1–70 Hz) and high (inset, 70–200 Hz) frequencies for in-trial (black) and inter-trial (red) periods.

Detailed data collection methods have been previously described (Gaidica et al., 2018), though all figures presented in this article represent new analyses of this data set. All animal procedures were approved by the Institutional Animal Care and Use Committee of the University of Michigan. Five adult male Long-Evans rats (Charles River Laboratories, Wilmington, MA, USA) were housed on a reversed light/dark cycle and food-restricted on training days. Operant chambers (ENV-009 Med Associates) were outfitted with five illuminated nose ports along one side with an opposite-facing reward port (Figure 1B). Rats were progressively trained to poke one of three illuminated center ports (only one port illuminated per trial) and then, after a variable delay (0.5–1 s, pulled from a uniform distribution), instructed to poke a neighboring port based on a brief low (1 kHz, “go left”) or high (4 kHz, “go right”) pitched tone. Correct trials were rewarded with a 45 mg sucrose pellet at the reward port. Rats were required to perform 80% of trials correctly for three sequential 1-h sessions before being implanted.

Electrophysiological implants were designed in SolidWorks and printed at the University of Michigan 3D Lab using biocompatible resins. Tetrodes spun from 12 μm wire (Sanvik PX000004) or 50 μm single wire electrodes (California Fine Wire) were interfaced with a Tucker Davis Technologies amplifier system (TDT, ZD64, AC32, PZ4, RZ2, and RS4) using a custom printed circuit board (Advanced Circuits). The entire electrode assembly was driven down with a precision drive screw. Immediately before surgery, the tetrodes (but not single wires) were gold plated according to a third-party protocol (Neuralynx), and impedances for all electrodes were recorded using a nano Z (White Matter) impedance tester. Tetrode impedances were near 200 kOhm and 50 μm wire impedances were near 80 kOhm at the time of implantation. All implants were surgically placed with the electrodes residing above the final recording site (Mthal; AP: −3.1 mm, ML: 1.2 mm, DV: −7.1 mm) with a ground and reference screw placed over the cerebellum contacting cerebral spinal fluid. The maximum inter-electrode distance was about 1.5 mm. Rats recovered for 1 week before retraining.

Electrodes were driven roughly 60 μm after each recording session. Wideband (0.1–10 kHz) neural signals were recorded at 24 kHz with the TDT system, which was interfaced with custom LabVIEW (RRID:SCR_014325) behavioral software to record behavior timestamps. The wideband signals were high-pass filtered in MATLAB (RRID:SCR_001622; 244 Hz to 6.10 kHz) and putative single-unit action potentials were extracted manually in Offline Sorter by matching waveforms and examining their auto- and cross-correlograms (Plexon; Gaidica et al., 2018). Potential duplicate units recorded across sessions were identified (Fraser and Schwartz, 2012); only one unit from each set of potential duplicates was included in the analysis (53 units were excluded on this basis).

### Statistics

**Figure 2:**
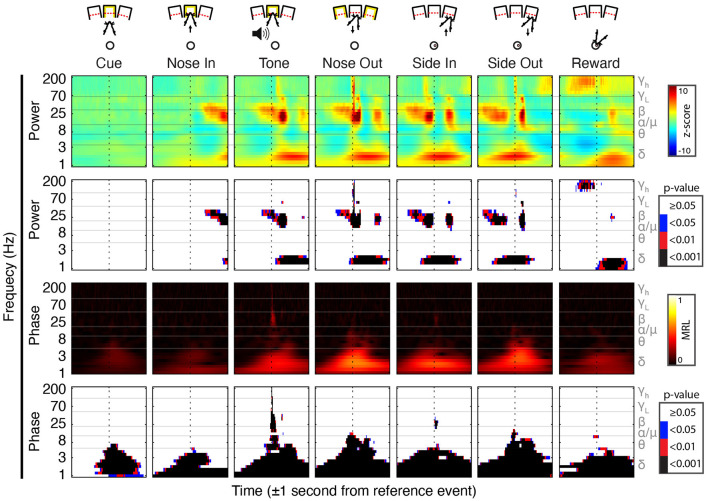
Peri-event field potential (FP) power and phase modulation. (Top Row) Behavioral schematic for a rightward-cued successful trial. (Second Row) Mean Gabor spectrograms for each event for all 30 sessions (*n* = 5 rats). (Third Row) *P*-values for the comparison of peri-event Z-scored power to the session-wide mean of Z-scored power. (Fourth Row) Mean resultant length (MRL) of event-locked FP phase. Higher values indicate time-frequency points at which the FP phase tends to be aligned across trials. (Bottom Row) Significance of peri-event phase alignment compared to the null hypothesis that phase is uniformly distributed at each peri-event time-frequency point.

The specific hypotheses being tested and methods for calculating significance are described in-line for each result and in the Statistics Summary in the Supplementary Materials. Specific *p*-values are provided where possible. In cases that require large numbers of comparisons (e.g., across multiple time-frequency combinations), *P*-value ranges are provided (e.g., Figures 2, 6). *P*-value ranges are also provided for shuffle tests, where the precision of *P*-value inferences is limited by the number of surrogate calculations performed. Such methods were used where standard statistical analyses were unavailable or the data structure was more amenable to bootstrapping techniques (for example, rats perform many sessions with variable numbers of trials). Phase calculations and statistics were made using the Circular Statistics Toolbox (CircStat, RRID:SCR_016651; Berens, 2009) for MATLAB (RRID:SCR_001622). When multiple comparisons were involved, results were corrected using the Bonferroni method. Although the Z-score is a statistical measure, it was sometimes converted to a *p*-value using a cumulative distribution function of the standard normal distribution.

**Figure 3:**
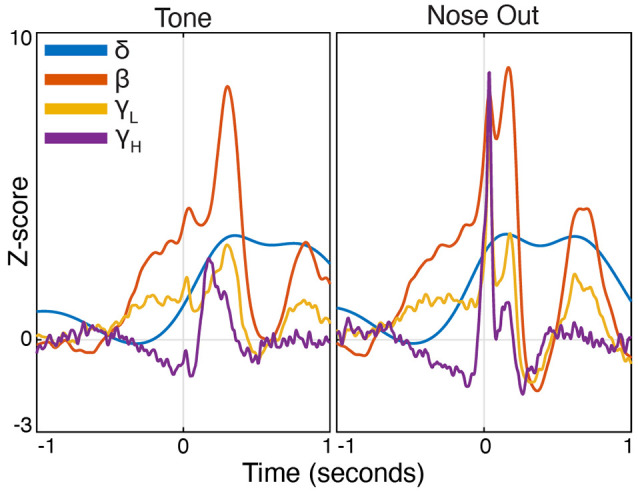
Peri-event FP power modulation in selected frequency bands at Tone and Nose-Out. Mean power modulation for all 30 sessions (*n* = 5 rats) in the delta, beta, low, and high gamma bands are all more tightly locked to Nose Out than Tone, suggesting a motor rather than sensory-related response.

**Figure 4:**
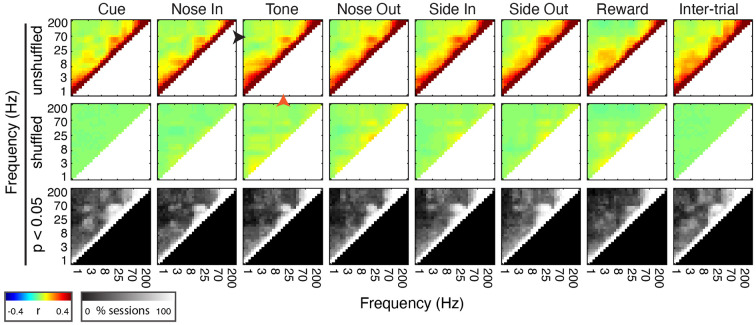
Mthal FP power in discrete frequency bands is comodulated during and between trials. (Top Row) Mean peri-event power-power comodulograms for each behavioral event and during the inter-trial interval for all 30 sessions (*n* = 5 rats). There is a consistent positive correlation between continuous beta (20 Hz, orange arrowhead at Tone) and low gamma (55 Hz, black arrowhead at Tone) power. (Middle Row) Comodulograms for the same events calculated using trial-shuffled data. (Bottom Row) Percent of sessions with significant power-power comodulation (*p* < 0.05, shuffle test—see “Materials and Methods” section) for each frequency pair.

**Figure 5:**
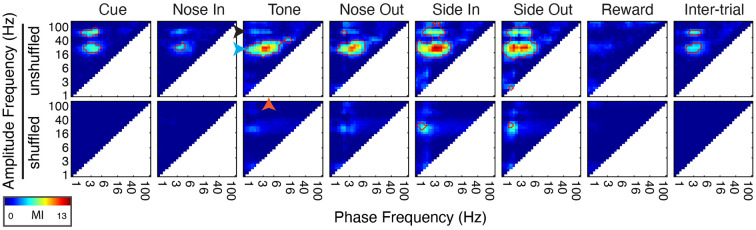
Phase-amplitude coupling (PAC) is dynamically modulated by task events. (Top) Peri-event PAC as assessed by the mean modulation index (MI, see “Materials and Methods” section) for all 30 sessions (*n* = 5 rats). Arrows indicate specific frequencies in the delta (2.5 Hz, orange arrowhead), beta (20 Hz, blue arrowhead), and low gamma (55 Hz, black arrowhead) bands. (Bottom) Same calculation using trial shuffled data. Red outlines highlight areas where PAC is significant (*p* < 0.05, corrected for multiple comparisons).

**Figure 6:**
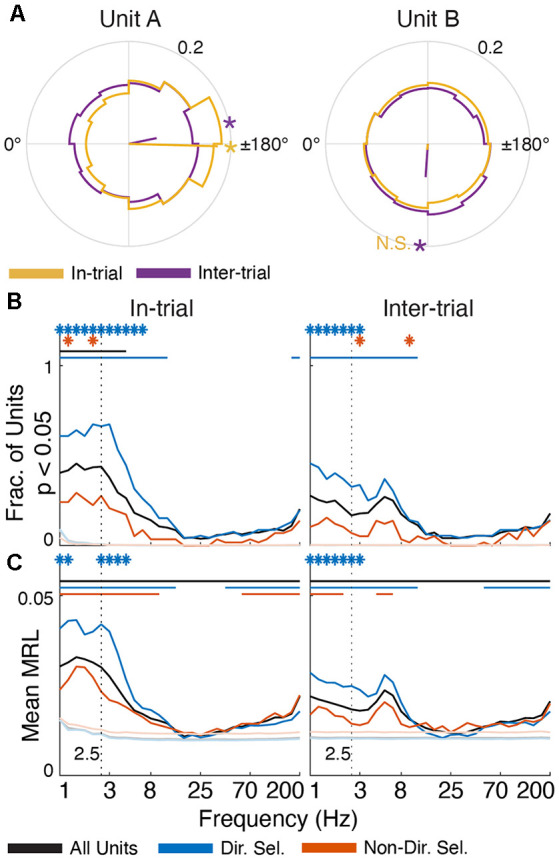
Single unit activity is selectively entrained in low-frequency oscillations. **(A)** Polar histograms (12 bins) of spike-FP phase alignment at 2.5 Hz for in-trial (yellow) and inter-trial (purple) epochs for two directionally-selective units. Unit A is subtly but significantly entrained during both epochs (MRL = 0.17, *p* = 6.65 × 10^−55^ in-trial; MRL = 0.06, *p* = 3.32 × 10^−6^ inter-trial). Conversely, Unit B is only significantly entrained to 2.5 Hz oscillations during the inter-trial epoch (MRL = 0.06, *p* = 1.69 × 10^−35^) but not the in-trial epoch (MRL = 0.01, *p* = 0.08). Yellow/purple lines indicate the mean resultant vector (MRV) for each unit during each epoch. Asterisks on the outer circles indicate MRV orientation for epochs of significant entrainment (“N.S.” indicates MRV orientation for non-significant entrainment). **(B)** The fraction of units from each population (all units in black, directionally selective units in blue, and non-directionally selective units in red) that were significantly entrained to FP oscillations across frequencies (*p* < 0.05, Rayleigh test for non-uniformity) during task engagement (“in-trial”) and the inter-trial interval (“inter-trial”). **(C)** Average MRL for each unit population across frequencies. The mean MRL for each population was significantly different from the surrogate spike trains at 2.5 Hz (*p* < 0.001). In panels **(B,C)** asterisks indicate frequencies at which directionally selective and non-directionally selective unit entrainment is significantly different from all units (*p* < 0.001). Solid lines indicate frequencies for which unit entrainment is significantly different from the firing-rate matched Poisson-distributed spike trains (*p* < 0.001).

### Code Accessibility

All data analysis was performed using custom MATLAB software which was routinely versioned using Git and made publicly available on GitHub^1^. Raw data are available upon request.

### Data Analysis

The wideband data were decimated by a factor of 16 (from 24 kHz to 1.5 kHz) using the MATLAB *decimate* function, which applies an anti-aliasing low pass filter before downsampling. Brief high-amplitude artifacts were identified as an instantaneous change in voltage greater than 2,000 μV and were then linearly interpolated to the next data point where the signal came back to within 50 μV of the pre-artifact amplitude. Forty-six high amplitude artifacts were removed from the recordings (44 between trials, two during trials). Raw trial data from the Cue to Reward event were converted to Z-scores using the mean and standard deviation from the whole session and the trial was removed if the absolute z-score exceeded five for 5% of the trial. Only two trials were eliminated by this criterion. Only correct trials were included in our analysis (*n* = 2,248 trials met inclusion criteria across 30 sessions). We used the same single unit population (*n* = 366) from a previous study that did not consider FP interactions with neuronal firing (Gaidica et al., 2018).

#### Power Spectrum

We visually inspected the raw data from all electrodes from each session and ranked their recording quality to select electrodes with no high amplitude artifacts. This enabled us to use a single, high-quality FP signal from each session for our analyses. Also, for spike-power and spike-phase correlations, we selected FP signals from wires where the spikes of interest were not recorded (though other units could be recorded on the FP wires), reducing the possible influence of the spike waveform itself on the FP.

We separately analyzed epochs during which the rat was engaged in the task (“in-trial,” between the Cue and Reward) and between trials (“inter-trial,” using a randomly-centered, trial time-matched segment that began after a Reward and before the next Cue). Therefore, the median trial length of 4.79 s is the same for the analyzed in-trial and inter-trial segments. We created the in-trial power spectrum by concatenating the wideband FP from all in-trial periods from a single session. Next, we performed a Fourier transform (*fft* in MATLAB) to obtain the power-frequency spectrum. To obtain an average spectrum for all sessions, we divided the spectrum by the average power of the 70–150 Hz segment, which accounted for variability associated with using different types of electrodes. We present the average spectrum using a conservative (0.2%) smoothing window (*smooth* in MATLAB, Figure 1C). We created the inter-trial power spectrum in the same way but used inter-trial segments.

#### FP Correlates of Behavior

A complex scalogram (1–200 Hz, 30 steps log-scale) was computed for each trial by applying a bank of Gabor filters to the raw data (Wallisch et al., 2013). Peri-event FPs (± 5 s) around each event were extracted and filtered, but only peri-event windows of ± 1 s or less were retained for the power, phase, and phase-amplitude coupling (PAC) analyses described below. This prevented filter edge effects from contaminating the analyses. FP power was calculated by taking the squared magnitude of the complex spectrum. For each session, we determined the mean (μ_baseline_) and standard deviation (*σ*_baseline_) of FP power from a surrogate distribution of power at randomly selected timestamps. To do this, we circularly shifted event timestamps by a random amount (from 0 to ± 2 s) to create 1,000 surrogate peri-event (± 1 s) scalograms (Canolty et al., 2007; Leventhal et al., 2012). The average μ_baseline_ and *σ*_baseline_ for each session (μ_session_ and *σ*_session_, respectively) allowed us to Z-score the peri-event power of each trial.

$$z_{\text{trial}}=\frac{power_{\text{trial}}-\mu_{\text{session}}}{\sigma_{\text{session}}}$$

FP phase was determined using the *angle* function in MATLAB on the complex scalogram. The mean resultant vector length (MRL) for phase data was computed using the *circ_r* function from CircStat. Z-scored power and the raw MRL values were calculated for each session and reported as the mean across sessions (Figures 2, 3). The significance matrix for FP power was generated by converting the Z-score mean power to *p-values* using a normal cumulative distribution function (*normcdf* in MATLAB), and phase significance was calculated using the Rayleigh test for non-uniformity on the phase angles from each trial over all the sessions, both with Bonferroni correction for multiple comparisons.

#### Power Comodulation

Power comodulograms were generated using the *corr* function in MATLAB (Pearson’s correlation). For each session, the power from all trials for each event (± 0.5 s) was concatenated. These time-series were used to calculate pair-wise power-power correlation coefficients for all frequency pairs (1–200 Hz, 30 steps log-scale). Trial-shuffled comodulograms were generated by pairing the FP power time series at frequency *f*_1_ from one trial with the power time series at frequency *f*_2_ from a randomly selected trial from the same session. This calculation was repeated for each *f*_1_ − *f*_2_ frequency pair 100 times for each session to generate surrogate comodulograms. Actual and surrogate comodulograms are presented as the average over all the sessions (Figure 4). For each session, *p*-values were calculated for each frequency pair as the fraction of trial-shuffled comodulograms with correlation coefficients greater than the real correlation coefficient.

#### Phase-Amplitude Coupling (PAC)

We quantified the strength of PAC using established methods (Canolty et al., 2006). A complex scalogram (1–200 Hz, 30 steps log-scale) was computed for peri-event time windows (± 0.5 s) for each trial. For each session, we concatenated data from all correct trials for each event. Thus, we obtained a complex time series for each event that was *n*-seconds long, where *n* is the number of trials in a session. We then obtained the time-series phase [*φ(t)*] by applying the *angle* function in MATLAB and amplitude [*A(t)*] by taking the magnitude of the complex time series. These data were used to determine the PAC between pairs of frequencies (*m*, *n*) across all events, with the constraint that the amplitude-frequency *m* was always greater than or equal to the phase frequency *n*. We achieved this by first creating a composite phase-amplitude signal (*z_t_*) from the session-wide time series data:

$$z{\left(t\right)}_{m,n}=A{\left(t\right)}_{m}e^{i\phi {\left(t\right)}_{n}}$$

The mean (*M_m,n_*) of *z(t)_m,n_* quantifies the deviation of *z(t)_m,n_* from a radially symmetric distribution of high-frequency FP amplitudes across low-frequency phases. To account for the possibility that *φ(t)_n_* is not uniformly distributed, we normalized *M_m,n_* for each session using 200 surrogates generated by adding a random lag τ to *A(t)*.

z(t+τ)m,n=A(t+τ)meiϕ(t)n

*M_surr_* is the mean of *z(t + *τ)*_m,n_* and is calculated separately for each surrogate phase-amplitude analysis. The mean (μ*M*_surr_) and standard deviation (σ*M_surr_*) of the surrogate distribution were calculated using *normfit* in MATLAB (where the input was all 200 *M_surr_* values). We report the modulation index (*MI_m,n_*) as the magnitude of the normalized *M_m,n_* (Figure 5).

$$MI_{m,n}=\left|\frac{M_{m,n}-\mu_{M_{surr}}}{\sigma_{M_{surr}}}\right|$$

A *p*-value was obtained for each phase-amplitude pair in the *MI* matrix using *normcdf* in MATLAB with the “upper” option to compute right-tailed probabilities. We corrected for multiple comparisons (Bonferroni method) by multiplying the *p*-values by the number of elements in *MI_m,n_* (*N* = 30 × 30). For example, using *α* = 0.05, the z-score contained in *MI* must exceed 3.87 to reach significance (determined using the *norminv* function in MATLAB on α ÷ N).

To determine if PAC was present independent of correlations between FP features and behavior, we recalculated surrogate MIs 1,000 times from a composite signal where the trial order of *A(t)* was shuffled (Stark and Abeles, 2005). This allowed us to generate a statistical measure for the fraction of shuffled *MI*s greater or less than the true *MI*.

#### Single Unit Entrainment

We extracted the instantaneous phase of the FP from the complex spectrum (using the MATLAB *angle* function) for each spike timestamp in equal duration in-trial and inter-trial periods. Next, we performed a Rayleigh test for the non-uniformity of circular data (CircStat *circ_rtest* function; Berens, 2009) for the compiled phases to obtain a *p*-value to reject the null hypothesis that spike timing is uniformly distributed from −180° to 180° (Figure 6). To determine if the number of units significantly entrained (*p* < 0.05) to each frequency was greater than chance, we generated firing rate matched, Poisson distributed spike trains for each unit and recalculated the *p*-values 1,000 times. We used the same data to calculate the mean MRL of the FP phase at each spike timestamp for each unit population (Figure 6C) and similarly compared it against Poisson spikes. *P-values* were determined as the fraction of significantly entrained unit percentages/MRL values from surrogate calculations that were greater than the actual value.

**Figure 7:**
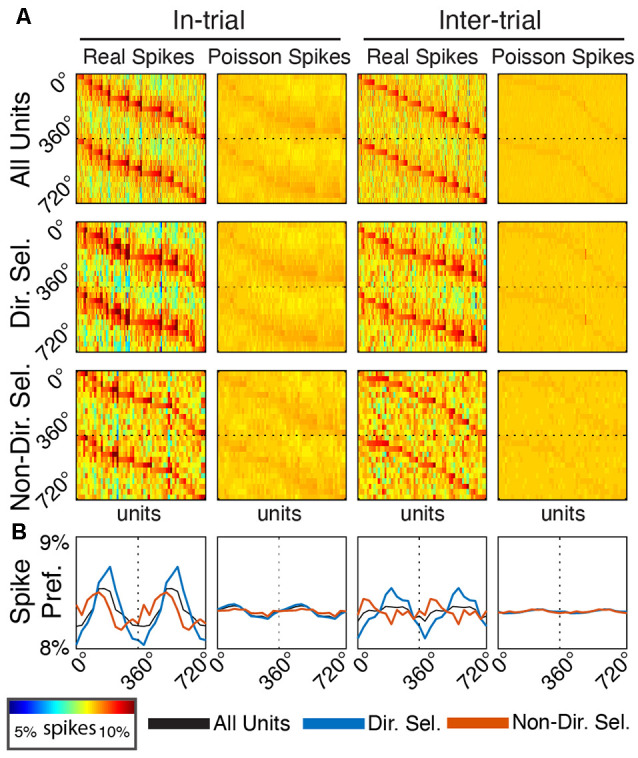
Single unit entrainment occurs at preferred delta phases specifically for directionally-selective units. **(A)** Spike-phase histograms for functionally-defined single unit populations and surrogate Poisson-distributed spike trains. Each column within the individual phase histograms represents a single unit. Colors indicate the percentage of spikes within a phase bin (12 bins from 0° to 360°, repeated to 720° for clarity). Units are sorted by their preferred phase separately for each plot. **(B)** Mean spike-phase histograms for each unit population and firing rate-matched surrogates.

To determine if the preferred firing phase was consistent across units, we generated spike histograms for each unit across 12 linearly-spaced phase bins between −180° and 180° (Figure 7). Each unit histogram was normalized by dividing each bin count by the total number of spikes for that unit to account for the spike rate. We used the same method described above to generate surrogate Poisson spike-phase histograms (*n* = 1,000), which were used to assess the significance of single unit phase preferences.

#### Spike-Power Cross-Correlations

**Figure 8:**
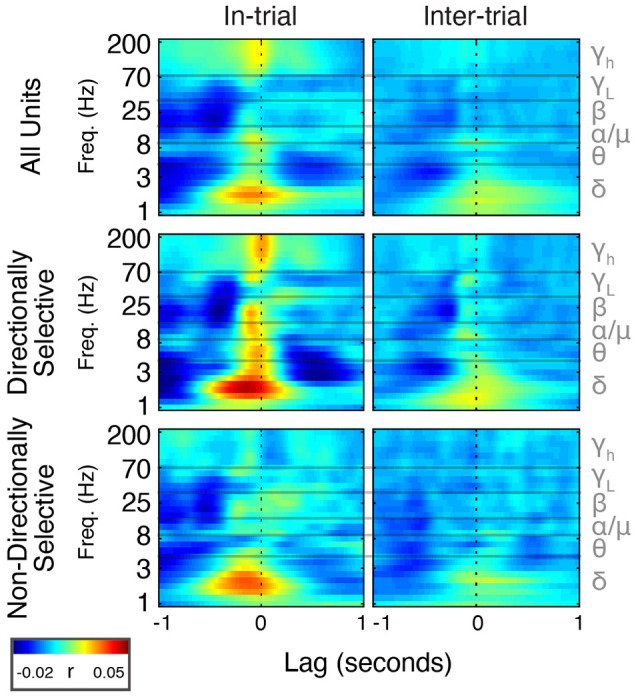
Spike timing is correlated with power modulation in specific frequency bands. Average cross-correlation between individual unit spike density estimates (SDEs) and FP power at different frequencies expressed as a heat map. *t* = 0 indicates a zero-lag correlation. Negative lags indicate that spikes lead FP changes, and positive lags indicate that FP changes lead spiking.

**Figure 9:**
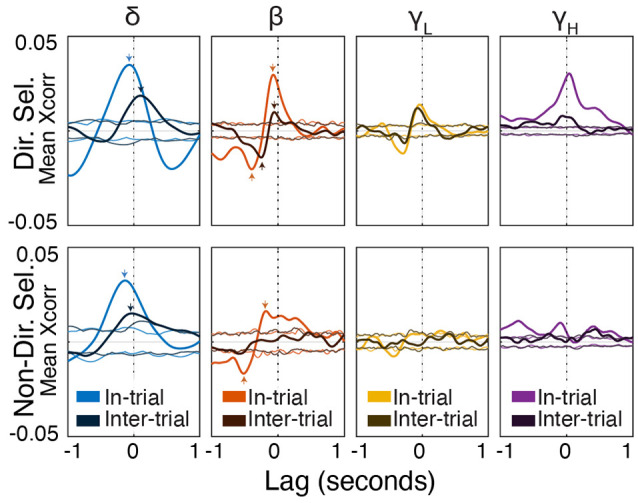
Beta/low-gamma power lags directionally-selective unit spiking during and between trials. Frequency band-specific cross-correlations (±1 s) for directionally selective (top) and non-directionally selective (bottom) units. Thick lines represent the actual cross-correlations; thin lines represent thresholds for significance at *p* < 0.01 (see “Materials and Methods” section). Small arrows indicate correlations mentioned in the “Results” section. Note that the cross-correlation patterns between directionally-selective units and beta/low gamma power are similar both during and between trials, which is not true for any other unit-frequency band combinations.

We used a cross-correlation to determine the relationship between FP power and single-unit activity for equal duration in-trial and inter-trial periods. First, we generated a session-wide continuous spike density estimate (SDE) for each unit and trial by convolving the vector of discrete spiking events with a 50 ms Gaussian kernel (Wallisch et al., 2013). Next, we extracted the relevant SDE segments for the in-trial and inter-trial periods. We cross-correlated these data with FP power (1–200 Hz, 30 steps log-scale) on a per-trial basis using the *xcorr* function in MATLAB with the “coeff” option so that the autocorrelations at zero lag equal 1. Cross-correlations are presented as the mean over all the trials and sessions (Figure 8). We recalculated each cross-correlation using a firing rate matched, Poisson distributed spike train 100 times, giving us a distribution of correlation values across time for each frequency. The maximum and minimum of that distribution are where we considered values to be significantly different from chance (Figure 9).

#### FP Correlates of Performance

**Figure 10:**
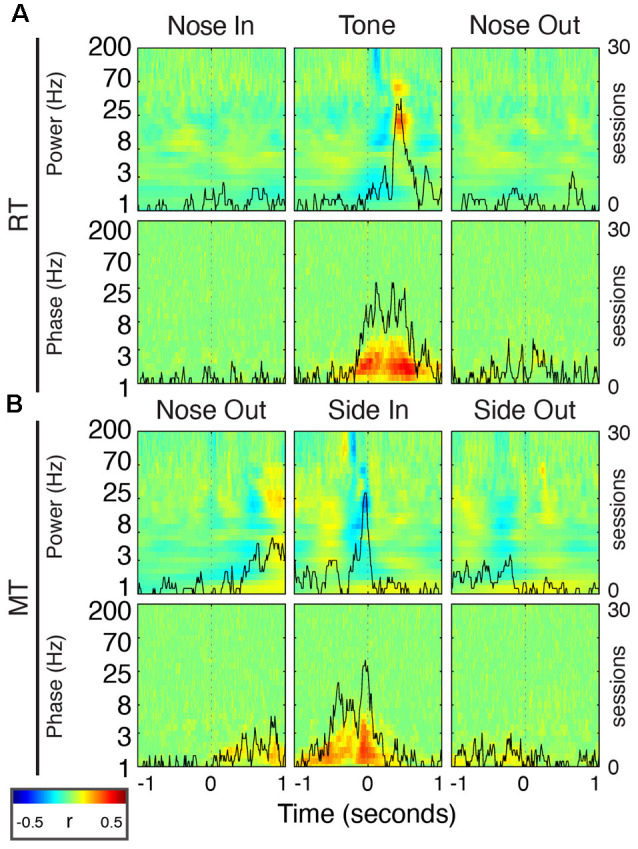
FP oscillations predict task performance. **(A)** Peri-event correlations between RT and FP power (top) or phase (bottom) for the Nose In, Tone, and Nose Out events. Correlation values are session-averaged. Solid black lines indicate the number of sessions that reached significance (*p* < 0.05) at each time point in the beta band (20 Hz) for power, and in the delta band (2.5 Hz) for phase. **(B)** Same as **(A)** for MT.

To determine relationships between FP features, RT, and movement time (MT), we used peri-event (±1 s) power and phase data for each frequency (1–200 Hz, 30 steps log-scale) and all trials. For each time point and frequency, we created a 1-by-*n* array of power (or phase) values, where, *n* was the number of trials in that session, along with a 1-by-*n* array of the RT (or MT) values for each trial. We used these two arrays as inputs to the *corr* function in MATLAB to calculate Spearman’s correlation coefficient for power-RT/MT, and the *circ_corrcl* function (CircStat toolbox, Berens, 2009) for phase-RT/MT correlations. Therefore, each time-frequency pair generated a single correlation coefficient and associated *p*-value between power/phase and RT/MT (Figure 10).

To determine if adjacent trials were consistently correlated either by physiological or performance metrics, we calculated the linear correlation coefficient between values of the *n*th and *n*th − *x* trial, where *x* = [1‥.10], for the mean z-scored delta power in a ± 0.5 s peri-event window (Cue and Nose Out events) as well as the trial RT (Supplementary Figure S2).

## Results

### FP Power and Phase Are Modulated by Task Performance in Discrete Frequency Bands

Rats (*n* = 5) were cued to immediately move left or right from a center nose port based on the pitch of an instructional cue (Figure 1, “Tone” event) until a high degree of accuracy was achieved (77 ± 17% over 30 sessions, mean ± SD). Reaction times (RT; the time from Tone to Nose Out) and movement times (MTs; the time from Nose Out to Side In) were consistent with similar studies (197 ± 10.3 ms and 302 ± 127 ms, respectively, mean ± SD, see Gaidica et al., 2018 for the full distributions; Dowd and Dunnett, 2005; Leventhal et al., 2012, 2014; Schmidt et al., 2013). The median trial duration was 4.79 s and the median inter-trial duration was 23.08 s. Similar to observations in the cortex (Murthy and Fetz, 1992; Saleh et al., 2010; Igarashi et al., 2013) and the basal ganglia (Berke et al., 2004; Masimore et al., 2005), the awake FP power spectrum in Mthal had discrete peaks in delta (1–4 Hz), theta (4–7 Hz), beta (13–30 Hz), and low gamma (30–70 Hz) bands (Figure 1C).

Task-linked Mthal FP power modulation was nearly identical to prior observations in the motor cortex and the basal ganglia during a similar task (Figure 2; Leventhal et al., 2012). Beta/low gamma power concurrently and transiently increased near the Nose Out and Side-Out events, contradicting the widely held view that beta power decreases with movement onset. This is likely explained by the delivery of instructive and imperative signals with the same stimulus—when these cues are separated, beta power increases during the inter-stimulus “hold” period and decreases with movement onset (including in our experiments; Donoghue et al., 1998; Saleh et al., 2010; Leventhal et al., 2012; Khanna and Carmena, 2017). The post-Nose Out beta/low gamma increase was more tightly locked to the Nose Out than the Tone event, suggesting that it is related to movement initiation rather than Tone perception (Figure 3). Furthermore, similar FP modulation at Side-Out argues against purely sensory modulation of beta/low gamma power. Delta power also increased at Nose Out and remained elevated through Side In. Finally, high gamma power transiently increased at Nose- and Side Out, and exhibited a sustained elevation as the rat moved from the nose ports to the food receptacle (Supplementary Figure S1).

To determine if the co-modulation of beta and low gamma power results from independent modulation of these frequency bands by the same behavioral events, we performed two additional analyses. First, correlations between beta and low gamma power were also present, albeit weaker, when the rats were not actively engaged in the task. Second, these correlations nearly, but not completely, disappeared when recalculated using trial-shuffled data (Figure 4). The persistence of beta-low gamma coupling in the trial-shuffled data is expected whether beta and low gamma oscillations are physiologically coupled to each other, or simply associated by independent correlations with the same behavioral events. However, if the association is purely due to independent behavior-FP correlations, there should be no difference between correlations in the trial-shuffled and real data. The significant decrease in beta-low gamma comodulation in the shuffled data argues that beta and low gamma oscillations are physiologically linked. Furthermore, the inter-trial persistence of beta-low gamma power comodulation argues that this is a general feature of Mthal physiology. We, therefore, refer to the “beta/low gamma” band in the rest of this manuscript, while acknowledging that there may be specific conditions under which beta and low gamma power could be uncorrelated.

In addition to FP power changes, the FP phase in specific bands was strongly modulated by the task. The beta/low gamma phase became sharply aligned at the Tone event (Figure 2), as previously observed in the basal ganglia (Leventhal et al., 2012). Phase alignment in the delta band was present as early as the Cue event and peaked at the Nose Out and Side-Out events. Collectively, these data suggest complex temporal coordination of FP power and phase in discrete frequency bands.

### Delta Phase Is Correlated With Beta and Low Gamma Power

The co-occurrence of a delta phase alignment and beta/low gamma power increase at Nose Out suggests that PAC is a prominent feature of Mthal physiology, as has been observed in other brain regions (Canolty et al., 2006; Tort et al., 2008; Cohen et al., 2009; Dejean et al., 2011; Belluscio et al., 2012; López-Azcárate et al., 2013). Indeed, delta-beta/low gamma PAC was significantly elevated throughout the task (“in-trial,” Figure 5), most prominently during movement from the Center to Side nose ports (i.e., Nose Out to Side In). Significant delta-beta/low gamma PAC was also present when the rat was not actively engaged in the task (“inter-trial”) and was greatly diminished when recalculated using trial-shuffled data. As for beta/low gamma amplitude-amplitude coupling, these results argue that delta-beta/low gamma PAC does not result simply from common responses to behavioral events.

### Delta Phase Is Correlated With Single Unit Mthal Activity

The phase of low-frequency oscillations was also correlated with the timing of single-unit activity (Lakatos et al., 2005; Fujisawa and Buzsáki, 2011; Crunelli et al., 2015), though the coupling strength was much smaller than in anesthetized rats (Nakamura et al., 2014). 59% of all units (*n* = 366) exhibited a non-uniform delta phase distribution during trials (black line in Figure 6B; defined as *p* < 0.05 for each unit, Rayleigh test for non-uniformity), which fell to 36% during the inter-trial period. These percentages were significantly greater than chance, as assessed by surrogate firing-rate matched Poisson spike trains. Furthermore, spike-phase entrainment was unique to the delta and theta bands. The average mean resultant length (MRL, a measure of phase uniformity; Leventhal et al., 2012; Wilson et al., 2018) of spike-FP phases across units was also significantly greater than chance for low frequencies (*p* < 0.001 at 2.5 Hz). These data support the notion that low-frequency oscillations subtly modulate Mthal single-neuron excitability in a behaviorally relevant manner.

We next investigated whether phase preferences differed for two functionally distinct subpopulations of Mthal units previously identified in this data set (Gaidica et al., 2018). Briefly, units responding strongly to Tone or Nose Out events were classified as “directionally” or “non-directionally” selective depending on whether their peri-Nose Out firing rates differed based on movement direction. “Directionally selective” unit activity was tightly linked to the Nose Out event and was correlated with which direction the rat would move, RT and MT. Conversely, “non-directionally selective” unit activity was more tightly locked to the Tone event and correlated with RT, but not MT or movement direction (366 total units, 103 directionally selective units, and 75 non-directionally selective units; these are the same populations identified in Gaidica et al., 2018).

These functionally defined populations were differentially entrained in delta oscillations. In-trial, 80% of directionally selective units were significantly entrained in the delta phase (Figure 6), which was the case for only 33% of non-directionally selective units. Between trials, delta entrainment decreased slightly for all units, resulting in entrainment for 57% of directionally selective units and 17% of non-directionally selective units. At higher (alpha/beta) frequencies, the entrainment for all three groups (directionally-selective, non-directionally selective, and all units) approached chance as assessed by surrogate Poisson spike trains. At frequencies greater than about 50 Hz, single units were again significantly entrained to the FP. This was weaker than at lower frequencies, with no to little difference between the directionally and non-directionally selective populations. The physiologic interpretation of this higher frequency entrainment is unclear but may reflect “leakage” of action potential spectra into the gamma band (Scheffer-Teixeira et al., 2013; Waldert et al., 2013). Because entrainment was assessed between spikes and FPs on different wires, this suggests that spike timing on the FP wire is (weakly) correlated with spike timing of the reference unit. In either case, such contamination is unlikely in the delta range where the strongest entrainment was seen (Waldert et al., 2013).

To determine if Mthal units tended to fire at the same preferred delta phase (assessed at 2.5 Hz), we created a spike-phase histogram for each unit (Figure 7). When rats were actively engaged in the task (“in-trial”), there was a clear phase preference for both directionally and non-directionally selective units (175.69°, *p* = 5.7 × 10^−7^ and 113.86°, *p* = 0.0044, respectively, Rayleigh test for non-uniformity). During the inter-trial period, the phase preference for non-directionally selective units was non-significant (*p* = 0.095). For directionally selective units, however, the phase preference persisted (191.13°, *p* = 9.5 × 10^−8^, Rayleigh test for non-uniformity) and was statistically indistinguishable from the in-trial phase preference (*p* = 1 compared with the in-trial phase, Kuiper two-sample test against the null hypothesis that the two distributions are identical). These results suggest that the in-trial phase entrainment observed for non-directionally selective units may be an artifact of two physiologic events (spiking and delta phase alignment) independently locked to the same behavioral event. Conversely, the phase entrainment of directionally selective units is more likely a pervasive feature of Mthal physiology.

### Directionally Selective Unit Activity Is Uniquely Correlated With FP Power

Delta phase is correlated with both beta/low gamma power and single-unit spiking. We, therefore, hypothesized that spiking and beta/low gamma power are also correlated. To test this, we cross-correlated FP power with a continuous SDE of Mthal single-unit activity and compared it to chance using firing-rate matched Poisson-distributed spike trains.

During trials, directionally selective unit activity was maximally correlated with beta power at a lag of −0.072 s (*r* = 0.03, *p* < 0.01). That is, changes in directionally selective unit firing rate tended to precede corresponding changes in beta power by 0.072 s (Figures 8, Figure 9). There was a smaller, yet significant negative correlation that peaked at −0.4 s (*r* = −0.02, *p* < 0.01), which may reflect decreased Mthal activity preceding the Side In the event when beta power is enhanced (see Gaidica et al., 2018, Figure 2). The cross-correlation pattern was strikingly similar during inter-trial intervals but attenuated (*r* = 0.01 at −0.058 s lag, *r* = −0.014 at −0.25 s lag, both *p* < 0.01), suggesting that beta power is enhanced following a “pause-fire” pattern of directionally selective Mthal unit spiking (Figure 9). Non-directionally selective unit activity was correlated with beta power slightly earlier, and to a lesser degree in-trial (*r* = 0.016 at *t* = −0.18 s lag, *r* = −0.017 at *t* = −0.52 s lag, both *p* < 0.01), but was not significantly correlated with beta power during the inter-trial period. These results suggest that the relationship between non-directionally selective unit activity and beta power resulted from independent correlations with behavioral events. Conversely, the relationship between directionally selective unit activity and beta power is likely a general feature of Mthal physiology.

Similar patterns were observed for directionally selective unit spike-low gamma power correlations, which were significant during both in-trial and inter-trial epochs. However, non-directionally selective unit activity was essentially uncorrelated with low gamma power during either epoch. The consistency of these correlations (or lack thereof) across both behavioral epochs supports the notion that directionally selective unit activity is uniquely linked to the FP.

The pattern of high gamma modulation during the task closely matched single unit Mthal activity patterns (Gaidica et al., 2018, Figure 2), consistent with observations that high-frequency oscillations are correlated with multi-unit activity. High gamma power best correlated with directionally selective unit activity, exhibiting roughly zero-lag between spiking and power increases. Therefore, as in cortex, Mthal high gamma power may serve as a surrogate for multi-unit activity (Ray et al., 2008; Manning et al., 2009; Watson et al., 2018).

Mthal single-unit activity also showed a small correlation with delta power, which was larger for directionally selective units. Unlike the beta- and low gamma-power correlations, the time lag and pattern of spike-delta power correlations were inconsistent between the in-trial and inter-trial periods (Figure 9). The peak spike-power correlation occurred at −0.068 s in-trial (*r* = 0.035, *p* < 0.01) but at 0.1 s during the inter-trial interval (*r* = 0.019, *p* < 0.01). Similar but smaller correlations were also observed for non-directionally selective units (*r* = 0.032 at −0.13 s in-trial, *r* = 0.015 at −0.03 s inter-trial, both *p* < 0.01).

In summary, the consistency of in-trial and inter-trial correlations argues for a unique physiological relationship between directionally selective unit activity and beta/low gamma power in Mthal.

### FP Correlates of Performance

Given the relationships between single-unit activity and task performance (Gaidica et al., 2018), and single-unit activity and FP features, we next examined relationships between FP features and task performance.

**Figure 11:**
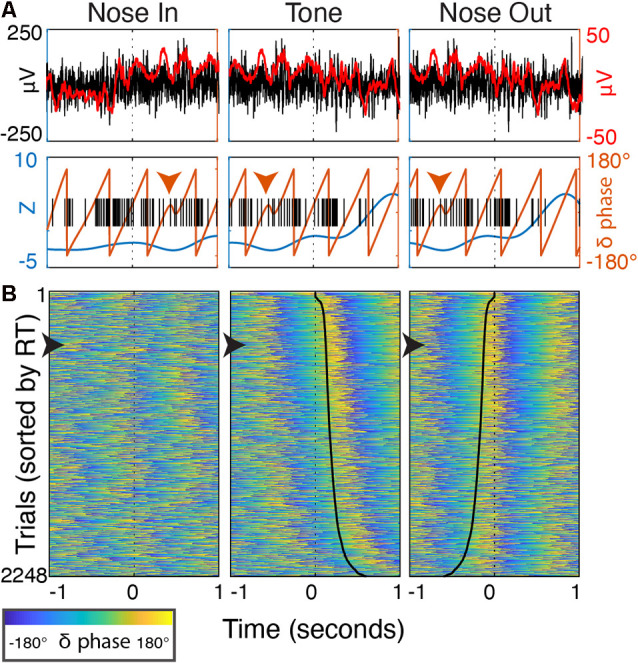
Delta phase evolution through individual trials. **(A)** Peri-event (±1 s) data from a single trial. Top—the unfiltered, wideband signal (black, left axis) with a 50 ms smoothing filter (red) highlighting discrete delta-band oscillatory patterns after Nose In and before Tone. Bottom—delta power (blue) and phase (orange line, orange arrowhead highlights phase discontinuity), and single-unit spike timing (black). **(B)** Peri-event delta phase from all trials over 30 sessions (*n* = 5 rats) sorted by RT. The trial from **(A)** is marked with a black arrowhead along the left border. Solid black lines in the Tone and Nose Out panels indicate the Nose Out and Tone events, respectively (i.e., the time from reference event to the black line is RT).

Delta phase near the Tone event was strongly correlated with RT (*p* < 0.05) in 19/30 recording sessions (Figure 10A; session-averaged *r* = 0.42 at *t* = 0.53 s after the event). This suggests that there is a preferred Mthal delta phase for movement initiation (Figures 2, 11) and that RT is (at least partially) determined by the distance from that preferred phase when the Tone plays (Lakatos et al., 2008). Neither delta power nor RT was consistently correlated across adjacent trials (Supplementary Figure S2), suggesting that the delta phase-RT correlation is not due to changes in attention modulating both delta oscillations and RT. While we cannot completely rule out the possibility that filtering propagates a delta phase reset at Nose Out back in time to the Tone event (de Cheveigné and Nelken, 2019), similar delta phase-RT correlations have been reported in a range of behavioral paradigms (Saleh et al., 2010; Stefanics et al., 2010; Hamel-Thibault et al., 2018). Furthermore, while phase discontinuities were occasionally observed in the filtered signal (Figure 11A, orange marker), they were not consistently present at the Nose Out event. Indeed, the delta phase varied smoothly from the Tone through Nose Out events across all trials (Figure 11B).

There was a similar delta phase correlation near the Side In event for MT (Figure 10B, *p* < 0.05 for 20/30 sessions; session-averaged *r* = 0.37 at *t* = 0.07 s before the event). However, since the delta phase was aligned at Nose Out, and MT was approximately the length of a single delta oscillation cycle, one would expect Side In to occur at different delta phases for different MTs. Thus, this delta phase-MT correlation does not represent a new finding independent of the Nose Out delta phase alignment.

Beta power was also correlated with RT in the peri-Tone period (*p* < 0.05 for 21/30 sessions; session-averaged *r* = 0.29 at *t* = 0.45 s after the event; Leventhal et al., 2012). As for the delta phase-MT correlation, however, this relationship can be explained by event-related beta modulation. For short RT, beta power after the Tone event increases earlier because, by definition, the Nose Out event is closer to the Tone event. We previously reported a small but significant correlation between striatal beta power and RT in the immediate pre-Nose Out period (Leventhal et al., 2012), but this finding was not replicated in Mthal (*p* < 0.05 in only 4/30 sessions). Whether this is due to subtle differences between basal ganglia and Mthal physiology, failure to detect a subtle correlation in the present study, or a false-positive result in the prior study, is unclear.

Finally, beta power was anticorrelated with MT just prior to Side In (*p* < 0.05 for 19/30 sessions; session-averaged *r* = −0.27 at *t* = 0.04 s before the event). However, because beta power increases transiently after Nose Out, beta power must be elevated just before Side In for short MT. This correlation is also, therefore, unlikely to represent a distinct effect from task-linked beta modulation. In summary, the delta phase at the Tone event was the only FP feature consistently and independently correlated with task performance.

## Discussion

We identified several interrelated correlations between Mthal FPs, Mthal single-unit activity, and behavior. First, FP phase in the delta band and power in multiple frequency bands (delta, beta, low, and high gamma) were modulated by specific behavioral events. Delta phase was strongly correlated with RT, beta/low gamma power, and single-unit spike timing. Given these correlations, it is not surprising that spike timing was also correlated with beta/low gamma power, though we did not find an independent relationship between beta power and RT. Most interestingly, Mthal single unit subpopulations previously identified based on behavioral correlations (Gaidica et al., 2018) exhibited distinct relationships with delta phase and beta power. Many of these correlations persisted during the intertrial interval, arguing that they do not arise simply from independent correlations with behavior. These findings unify prior observations of correlations between the delta phase, beta power, and behavior. They also provide new insights into how motor system FP oscillations may be generated and linked to behavior.

Beta oscillations are suggested to represent a stabilized network state during which motor plans are less likely to change (Gilbertson et al., 2005; Pogosyan et al., 2009; Engel and Fries, 2010; Khanna and Carmena, 2017), which may serve the adaptive purpose of preventing distractors from interfering with a recently adopted plan. This interpretation is supported by small, but significant and reproducible, correlations between beta power and RT (Leventhal et al., 2012; Khanna and Carmena, 2017; Shin et al., 2017; van Wijk, 2017; Torrecillos et al., 2018). However, we did not replicate that finding. This could be due to differences in recording sites, as prior correlations were found in basal ganglia or cortex. However, patterns of event-related beta power modulation were nearly identical in striatum, cortex, and Mthal (Leventhal et al., 2012), making it less likely that Mthal and cortical-basal ganglia beta oscillations have distinct physiologic interpretations concerning RT. We suggest instead that beta power is linked to RT indirectly *via* delta-beta PAC, explaining why weak beta-RT correlations are frequently observed.

Delta phase was more strongly and consistently correlated with RT before movement onset than beta power. While we cannot exclude the possibility that the task-related increase in delta power represents an event-related potential (ERP), several factors suggest that ongoing delta oscillations modulate task performance. First, delta phase becomes correlated with RT well before Nose Out. This implies a preferred phase of ongoing delta rhythms for movement onset, rather than delta phase resetting at Nose Out. Consistent with this argument, delta phase evolves continuously at Nose Out (Figure 11) without consistent phase-resets. Finally, delta phase is correlated with single-unit spiking both during and between trials, arguing that a physiologically meaningful delta rhythm is present even outside of task performance.

Similar delta phase-RT correlations have been found during tasks in which cortical delta oscillations entrain to rhythmic stimuli (Lakatos et al., 2008; Stefanics et al., 2010; Arnal et al., 2015). FP oscillations may modulate neuronal excitability through ephaptic interactions (Anastassiou et al., 2010; Tiganj et al., 2014), or simply reflect aggregate synaptic drive that influences spiking probability (Pesaran et al., 2018). In either case, active entrainment of FP oscillations to rhythmic cues is a potential mechanism to optimize neuronal excitability at the time of anticipated salient stimuli (Schroeder and Lakatos, 2009). It remains unclear, however, whether such mechanisms are generalizable to single interval timing (Breska and Deouell, 2017; Hamel-Thibault et al., 2018; Zoefel et al., 2018). In our task, instructive/imperative cue (Tone) timing is somewhat predictable, occurring 0.5–1.0 s after Nose In. The presence of increased delta phase coherence across trials even before Nose In (Figure 2), and the smooth progression of delta phase at Nose Out (as opposed to an abrupt phase reset, Figure 11), support the idea that delta phase actively aligns to increase the probability that the Tone arrives at a favorable phase for quick reactions.

A plausible mechanism for delta phase-RT correlations is that the delta phase predicts (perhaps influences) Mthal spike timing, which drives motor cortex to initiate movement. This could be explained by the FP reflecting synchronized inputs to Mthal, which modulate local excitability. Thus, if the Tone arrives just after the optimal phase, a full delta cycle would have to repeat before Mthal neurons are maximally excitable. In support of this hypothesis, units whose activity was correlated with RT were strongly entrained in delta rhythms. A related but slightly different interpretation is that delta oscillations reflect cortical excitability, with cortical neurons more likely to fire at specific delta phases and drive phase-locked firing of thalamic neurons (Lakatos et al., 2005; Rule et al., 2018).

Our data also suggest a mechanism for delta-beta PAC. Delta phase is correlated with Mthal single-unit spike timing which in turn is correlated with, and possible causes, cortical beta oscillations that are propagated throughout basal ganglia-thalamocortical circuits (Jones et al., 2009; Sherman et al., 2016; Reis et al., 2019). Such a model would explain the small frequently observed correlations between beta power and RT, as well as associations between “bursty” Mthal activity and beta oscillations in Parkinson Disease (Kühn et al., 2009; Ellens and Leventhal, 2013; Devergnas et al., 2015; Reis et al., 2019). If delta phase-modulated Mthal single-unit activity both initiates movement and drives cortical beta oscillations, one would expect weak correlations between beta power and RT. This model does not exclude the possibility that other sources of beta oscillations (e.g., intrinsic basal ganglia oscillators, McCarthy et al., 2011; Tachibana et al., 2011; Mirzaei et al., 2017) are independently associated with the behavior.

The identity of directionally- and non-directionally selective units has important implications for understanding subcortical mechanisms of motor control, as well as how FP oscillations are generated and regulated. One possibility is that these functionally-defined units are anatomically defined by layer-specific cortical projections. Thalamic afferent activity in motor cortical layer 1 is correlated with the speed of individual lever pulls performed by mice, and layer 3 afferents are active at movement initiation (Tanaka et al., 2018). These patterns are strikingly similar to our directionally- and non-directionally selective units, respectively (Gaidica et al., 2018). Furthermore, modeling studies suggest that coordinated layer-specific thalamocortical inputs drive cortical beta oscillations (Sherman et al., 2016). This model requires precisely-timed layer 1 input, which could be provided by directionally-selective units given the correlation between their activity and beta oscillatory power.

A related possibility is that directionally- and non-directionally selective units reside in basal ganglia- and cerebellar-recipient Mthal, respectively. Mthal comprises two mostly non-overlapping subregions defined by basal ganglia or cerebellar afferents (Deniau et al., 1992; Kuramoto et al., 2011) that tend to project to cortical layers 1 and 3/5, respectively (though not with 100% certainty; Herkenham, 1980; Kuramoto et al., 2009, 2015; Tanaka et al., 2018). In addition to the evidence suggesting that functionally defined Mthal units may have layer-specific projections, several observations also suggest that directionally-selective units reside in basal ganglia-recipient Mthal. First, directionally selective unit activity is correlated with features of task performance commonly attributed to the basal ganglia (action selective and movement vigor). Indeed basal ganglia manipulations influence RT, MT, and movement direction in nearly identical tasks (Carli et al., 1985; Dowd and Dunnett, 2005; Leventhal et al., 2014). Second, directionally selective units were consistently entrained to delta oscillations during wakefulness, as are basal ganglia-recipient Mthal units under anesthesia (Nakamura et al., 2014). Finally, directionally-selective unit activity is correlated with beta oscillatory power, which is associated with basal ganglia-thalamocortical circuitry (Leventhal et al., 2012; López-Azcárate et al., 2013; Brittain and Brown, 2014; Feingold et al., 2015).

One limitation of this study is that the *in vivo* geometry of the electrodes was inconsistent, making it difficult to localize FP origins. However, modeling studies and previous recordings provide clues to potential FP sources. Sources for delta, beta, and gamma oscillations have been identified in neocortex using linear electrode arrays (Kandel and Buzsáki, 1997; Lakatos et al., 2008; Cardin et al., 2009; Torres et al., 2019), and modeling studies suggest that cortical FPs can spread several millimeters into subcortical structures (Torres et al., 2019). We may, therefore, have recorded cortical FPs volume-conducted into Mthal. This is supported by consistent delta-beta PAC in cortical recordings (Saleh et al., 2010; Arnal et al., 2015), and would suggest that direct FP-single unit interactions occur in cortex, not Mthal. However, locally-referenced electrodes have also detected similar cross-frequency coupling in the striatum and the subthalamic nucleus (López-Azcárate et al., 2013). Striatal origins for delta, beta, and gamma oscillations have also been proposed (Chartove et al., 2020), and subthalamic nucleus-globus pallidus, pars externa connections are implicated in generating beta oscillations (Tachibana et al., 2011; Mirzaei et al., 2017). Finally, modeling studies suggest that focal synaptic input to nonlaminar subcortical structures (e.g., striatum and thalamus) can generate measurable LFPs (Tanaka and Nakamura, 2019). It is therefore likely that the recorded FPs result from complex combinations of synaptic currents originating in multiple structures (Herreras, 2016). To better localize the origins of Mthal FPs, recordings with orderly arrays of electrodes will be needed.

In summary, we found complex relationships between Mthal FP oscillations, single-unit activity, and performance of a two-alternative forced-choice task. These results support a model in which low-frequency FP oscillations either modulate or reflect Mthal neuronal excitability, which in turn drives movement initiation and regulates higher frequency (beta/low gamma) oscillations. These results potentially explain consistently observed correlations between delta phase, beta power, and behavior. A critical open question is the identity of functionally distinct Mthal neuronal populations, which we predict receive distinct subcortical afferents and project to different cortical layers. These predictions have significant implications for understanding subcortical contributions to motor control and should be testable by combining modern anatomic tracing techniques with high-density electrophysiology and/or optogenetics.

## Data Availability Statement

The raw data supporting the conclusions of this article will be made available by the authors, without undue reservation.

## Ethics Statement

The animal study was reviewed and approved by Institutional Animal Care and Use Committee, University of Michigan.

## Author Contributions

MG and DL designed the experiments, analyzed and interpreted the data and wrote and revised the manuscript. MG, AH, and CC performed the experiments.

## Conflict of Interest

The authors declare that the research was conducted in the absence of any commercial or financial relationships that could be construed as a potential conflict of interest.

## Footnotes

^1^https://github.com/leventhallab
