# Supplementary material for: Interactions Between Motor Thalamic Field Potentials and Single-Unit Spiking Are Correlated With Behavior in Rats
Source: Front Neural Circuits. 2020 Aug 13;14:52. doi: 10.3389/fncir.2020.00052 (PMC7457120; doi:10.3389/fncir.2020.00052)
Supplement: Supplementary file 1 [file Table_1.DOCX]

Supplementary Material

**Supplementary Figure 1.** Peri-event power modulation in delta, beta, low, and high gamma bands extended to ±8 s, so that the entire task structure is included in each plot (2,248 trials over 30 sessions). Z-scores were determined using a bank of 1,000 surrogates for each trial where event times were shifted by random amounts pulled from a uniform distribution between ±2 s. Colored dots along the bottom x-axis indicate where z-scores are significant (p < 0.05) determined using a normal cumulative distribution function with Bonferroni correction for multiple comparisons.

**Supplementary Figure 2.** Correlations between the value of each previous 10 trials within the same session and the current trial for delta power (Cue and Nose Out events) and reaction time (RT). Solid black line – average correlations. Gray lines indicate correlations for each session (n = 30). Red markers indicate where the pairwise linear correlation (*corr()* function in MATLAB) was significant (p < 0.05).

**Statistics Summary**

**Figure 1.** None.

**Figure 2.** Peri-event (± 1 s) power and phase were extracted from the FP around each event (controlling for edge effects). FP power was calculated by taking the squared magnitude of the complex spectrum and Z-scored using a bank of 1,000 surrogates. The significance matrix for FP power was generated by converting the Z-score mean power to p-values using a normal cumulative distribution function (*normcdf* in MATLAB) with Bonferroni correction for multiple comparisons. FP phase was determined using the *angle* function in MATLAB on the complex scalogram. The mean resultant vector length (MRL) for phase data from all trials was computed using the *circ_r* function from CircStat with Bonferroni correction for multiple comparisons.

**Figure 3.** None.

**Figure 4.** Pair-wise power-power correlation coefficients for all frequency pairs were generated using the *corr* function in MATLAB (Pearson’s correlation). 100 trial-shuffled comodulograms were generated by pairing frequency pairs from different, random trials and was used to evaluate significance (p < 0.05) for each session.

**Figure 5.** Time-series phase and amplitude values for all successful trials were concatenated into a single, composite phase-amplitude signal. A modulation index (*MI*) was computed for each frequency pair based on a normal fit (*normfit()*) to the distribution of 200 surrogates composed of randomly time-lagged data from the composite time-series. The mean of the composite signal was normalized using the distribution mean and standard deviation, and its magnitude is the *MI* for that frequency pair. A p-value was obtained for each phase-amplitude pair in the *MI* matrix using *normcdf()* in MATLAB for right-tailed probabilities and corrected for multiple comparisons using the Bonferroni method.

To determine if PAC was present independent of correlations between FP features and behavior (bottom row), we recalculated surrogate MIs 1,000 times where the trial order was shuffled. This allowed us to generate a statistical measure for the fraction of shuffled *MI*s greater or less than the true *MI*.

**Figure 6.** A Rayleigh test for non-uniformity of circular data (CircStat *circ_rtest* function) was used to generate a p-value that rejected the null hypothesis that spike timing is uniformly distributed from -180° to 180°.

To determine if the number of units entrained and MRL was significant (p < 0.05) for each frequency, we generated 1,000 firing rate matched, Poisson distributed spike trains whereby p-values were determined as the fraction of significantly entrained unit percentages/MRL values from surrogate calculations that were greater than the actual value.

**Figure 7.** Spike histograms for each unit across 12 linearly-spaced phase bins between -180° and 180° were generated and normalized by dividing each bin count by the total number of spikes for that unit to account for spike rate. We used the same method as described in Figure 6 to generate surrogate Poisson spike-phase histograms (n = 1,000), which were used to assess the significance of single unit phase preferences.

**Figure 8.** None.

**Figure 9.** A session-wide continuous spike density estimate (SDE) for each unit and trial was generated and SDE segments for the in-trial and inter-trial periods were extracted, then cross-correlated with FP on a per-trial basis using the *xcorr* function in MATLAB such that the autocorrelations at zero lag equal 1. The mean of these values is presented in thick, colored lines. We recalculated each cross-correlation using a firing rate matched, Poisson distributed spike train 100 times, giving us a distribution of correlation values across time for each frequency. The maximum and minimum of that distribution are where we considered values to be significantly different from chance, presented as thin, colored lines.

**Figure 10.** The *corr* function in MATLAB was used to calculate Spearman’s correlation coefficient for power-RT/MT, and the *circ_corrcl* function (CircStat toolbox) for phase-RT/MT correlations. Therefore, each time-frequency pair generated a single correlation coefficient and associated p-value between power/phase and RT/MT.

**Figure 11.** None.

**Figure 12.** None.
